# The Role of Mucosal Immunity in Pertussis

**DOI:** 10.3389/fimmu.2018.03068

**Published:** 2019-01-14

**Authors:** Luis Solans, Camille Locht

**Affiliations:** ^1^Center of Infection and Immunity of Lille, Institut Pasteur de Lille, Lille, France; ^2^Inserm U1019, Lille, France; ^3^CNRS UMR8204, Lille, France; ^4^Center for Infection and Immunity of Lille, Univ. Lille, Lille, France

**Keywords:** pertussis, secretory IgA, tissue-resident memory T cells, mucosal vaccine, live attenuated vaccine

## Abstract

Pertussis or whooping cough, mainly caused by *Bordetella pertussis*, is a severe respiratory disease that can affect all age groups but is most severe and can be life-threatening in young children. Vaccines against this disease are widely available since the 1950s. Despite high global vaccination coverage, the disease is not under control in any country, and its incidence is even increasing in several parts of the world. Epidemiological and experimental evidence has shown that the vaccines fail to prevent *B. pertussis* infection and transmission, although they are very effective in preventing disease. Given the high infection rate of *B. pertussis*, effective control of the disease likely requires prevention of infection and transmission in addition to protection against disease. With rare exceptions *B. pertussis* infections are restricted to the airways and do not usually disseminate beyond the respiratory epithelium. Therefore, protection at the level of the respiratory mucosa may be helpful for an improved control of pertussis. Yet, compared to systemic responses, mucosal immune responses have attracted relatively little attention in the context of pertussis vaccine development. In this review we summarize the available literature on the role of mucosal immunity in the prevention of *B. pertussis* infection. In contrast to vaccination, natural infection in humans and experimental infections in animals induce strong secretory IgA responses in the naso-pharynx and in the lungs. Several studies have shown that secretory IgA may be instrumental in the control of *B. pertussis* infection. Furthermore, studies in mouse models have revealed that *B. pertussis* infection, but not immunization with current acellular pertussis vaccines induces resident memory T cells, which may also contribute to protection against colonization by *B. pertussis*. As these resident memory T cells are long lived, vaccines that are able to induce them should provide long-lasting immunity. As of today, only one vaccine designed to induce potent mucosal immunity is in clinical development. This vaccine is a live attenuated *B. pertussis* strain delivered nasally in order to mimic the natural route of infection. Due to its ability to induce mucosal immunity it is expected that this approach will contribute to improved control of pertussis.

## Introduction

Whooping cough, also referred to as pertussis, is a severe respiratory disease that can be life threatening in newborns and non-vaccinated young children. The disease can also occur in older children, adolescents and adults. Although it is usually not fatal in these age groups, it represents a risk of various serious complications, including pneumothorax, and rib fractures (1). The main causative agent of whooping cough is *Bordetella pertussis* (2), a gram negative coccobacillus which is able to colonize the human upper respiratory tract by attaching to the ciliated cells. Other *Bordetella* species, such as *Bordetella parapertussis* (3) and *Bordetella holmesii* (4), can cause diseases similar to pertussis, albeit usually with much less severe symptoms than typical whooping cough caused by *B. pertussis*.

It is estimated that whooping cough causes globally around 200,000 deaths per year and more than 24 million new pertussis cases in children younger than 5 years were reported in 2014 (5), in spite of the wide usage of efficacious pertussis vaccines (6). Although the pertussis incidence has dramatically decreased since the first introduction of these vaccines (7), whooping cough remains a major global public health problem, mostly in resource-poor countries. However, surprisingly, its prevalence is also strongly increasing in westernized countries (8), especially since the switch from the first-generation, whole-cell vaccines to the new-generation, acellular pertussis vaccines. Several reasons may account for this resurgence, including faster waning of immunity through acellular compared to whole-cell vaccines and potential strain adaptation to escape vaccine-induced immunity (9). Although the use of the current pertussis vaccines have certainly led to a spectacular reduction in whooping cough disease incidence, the vaccines did not appear to interrupt the circulation of *B. pertussis* in susceptible populations, and several studies have shown that symptom-less carriage of *B. pertussis* in adults is more common than previously appreciated (10–14). In fact, asymptomatic transmission may be a major driver of the resurgence of pertussis in highly vaccinated populations, as suggested by mathematical modeling studies (15). Studies with non-human primates have shown that both the whole-cell and acellular vaccines provide strong protection against pertussis disease, but none of them prevents infection by *B. pertussis* (16). Therefore, it seems logical to assume that optimal control of pertussis requires the use of vaccines that prevent both whooping cough disease and infection by *B. pertussis*. Only vaccines that prevent or strongly limit infection of a causative infectious agent will induce sufficient herd immunity to eventually eradicate the respective disease.

## Mucosal Immune Responses Induced by *B. Pertussis* Infection

### *B. pertussis* as a Strictly Mucosal Pathogen

*B. pertussis* is known to be mainly an upper respiratory tract pathogen, but lower respiratory tract infections can also occur, especially in severe pertussis cases (2). However, dissemination outside of the respiratory tract is almost unheard of. Disseminated *B. pertussis* infection has only rarely been described and has been seen exclusively in severely immune-compromised individuals (17). In mice *B. pertussis* also remains in the respiratory tract and disseminates to other organs, such as liver and spleen, only in mice with severe immune defects, such as IFN-γ-receptor deficient mice (18). It is therefore likely that the local immunity in the respiratory tract may be important for the control of *B. pertussis* infection. Yet, today, all commercially available vaccines are given parenterally and do not induce local immune responses. Systemic immune responses have been extensively studied in several models [summarized in the review by (19)], whereas comparatively little is known about the role of local immune responses in the control of whooping cough. Unlike immunization with current pertussis vaccines, infection with *B. pertussis* appears to induce sterilizing immunity in the airways of non-human primates (16) and it is conceivable that this is linked to a potent mucosal immune response to the infection. Strong IL-17 induction was seen in the nasopharyngeal washes 5–7 days after *B. pertussis* infection of baboons (20). This was paralleled by the strong induction of IL-6 and IL-23 in convalescent baboons and followed by the increase in Th-17-associated chemokines and cytokines, such as GCSF, important for neutrophil differentiation, IL-8, MCP-1, and MIP-1a, important for the regulation of influx of various cell types involved in the clearance of respiratory pathogens. In mice IL-17 has been shown to also play an important role in the production of secretory IgA (sIgA) in the mucosal lumen by the induction of the poly-Ig receptor on the basal side of the epithelial cells (21, 22) and by facilitating the recruitment of B cells upon infection (22). However, mucosal IgA responses could not be measured in the nasopharyngeal washes of *B. pertussis*-infected baboons, due to the lack of suitable antibodies that can recognize baboon IgA. The source of the mucosal IL-17, innate cells or CD4^+^ Th17 cells, has also yet to be determined in the convalescent baboons.

### Secretory IgA Responses Induced by Infection With *B. pertussis*

In humans *B. pertussis* infection has long been shown to lead to potent anti-*B. pertussis* IgA production in nasal secretions (23). They appear during week 2–3 of illness and can often still be found when the *B. pertussis* organism can no longer be recovered. They can persist for several months after the onset of symptoms, but usually decline to low levels after 6 months. Anti-*B. pertussis* sIgA from nasal washes and serum IgA of convalescent patients have been shown to inhibit adherence of *B. pertussis* to human respiratory epithelial cells (24). These antibodies are preferentially induced during convalescence and much less so after vaccination. In addition to inhibiting *B. pertussis* adherence to epithelial cells, anti-*B. pertussis* IgA from convalescent subjects can also enhance *B. pertussis* uptake by human polymorphonuclear leukocytes *in vitro* via the myeloid IgA receptor FcαRI (CD89) and lead to subsequent bacterial killing (25). However, this study was done using serum IgA, and it remains to be seen whether similar observations can be made with sIgA.

The effect of IgA on the course of infection by *B. pertussis* has been investigated by the use of IgA-deficient mice (26). Surprisingly, when wild type or IgA-deficient mice were intranasally challenged with 5 × 10^5^ colony-forming units (CFU) of virulent *B. pertussis*, similar amounts of CFUs were recovered for both mouse strains from the lungs, trachea, and nasal cavities at each time point, up to 105 days post-inoculation, suggesting that IgA plays no critical role in clearing *B. pertussis* from the murine respiratory tract. Furthermore, a first infection by *B. pertussis* appeared to protect wild-type and IgA-deficient mice alike against a secondary infection in the lungs, trachea, and the noses, suggesting that IgA is not required neither to prevent a secondary infection of the upper and lower respiratory tract of *B. pertussis*-primed mice. Interestingly, this is in contrast to *Bordetella bronchiseptica* infections, for which IgA appeared to play a critical role in protection of the upper, but not of the lower respiratory tract. However, these findings are in conflict with recent data obtained from mice vaccinated with BPZE1, a live attenuated nasal pertussis vaccine (27). BPZE1 is a *B. pertussis* TohamaI derivative containing mutations in its genome that eliminate or inactivate three major *B. pertussis* toxins: tracheal cytotoxin, dermonecrotic toxin, and pertussis toxin (PTx) (28). In contrast to vaccination with acellular pertussis vaccines, nasal administration of BPZE1 protects mice against nasal as well as lung colonization by virulent *B. pertussis*, and BPZE1-induced protection in the nose was strongly diminished in IgA-deficient mice (27). The reasons for these conflicting findings are not clear, but the most important difference between the two studies is the use of a virulent *B. pertussis* in the former and of an attenuated strain in the latter study. As the attenuated strain lacks two toxins and produces inactive PTx, could it be that one or several of these toxins produced by the virulent strain overrides the need for IgA? Whether this or other technical differences between the two studies may explain the different outcomes between them remains to be investigated.

In humans, there is no published evidence so far that IgA deficiency may be associated with enhanced *B. pertussis* infection. However, epidemiological studies addressing specifically *B. pertussis* infection are scarce, and most studies focus on pertussis disease. It may thus very-well be possible that IgA deficiency increases the likelihood of being infected by *B. pertussis*, without necessarily impacting on pertussis disease, against which other immune mechanisms, such as PTx neutralization by serum IgG, may be at play.

### Local Cellular Responses Induced by Infection With *B. pertussis*

**Figure 1:**
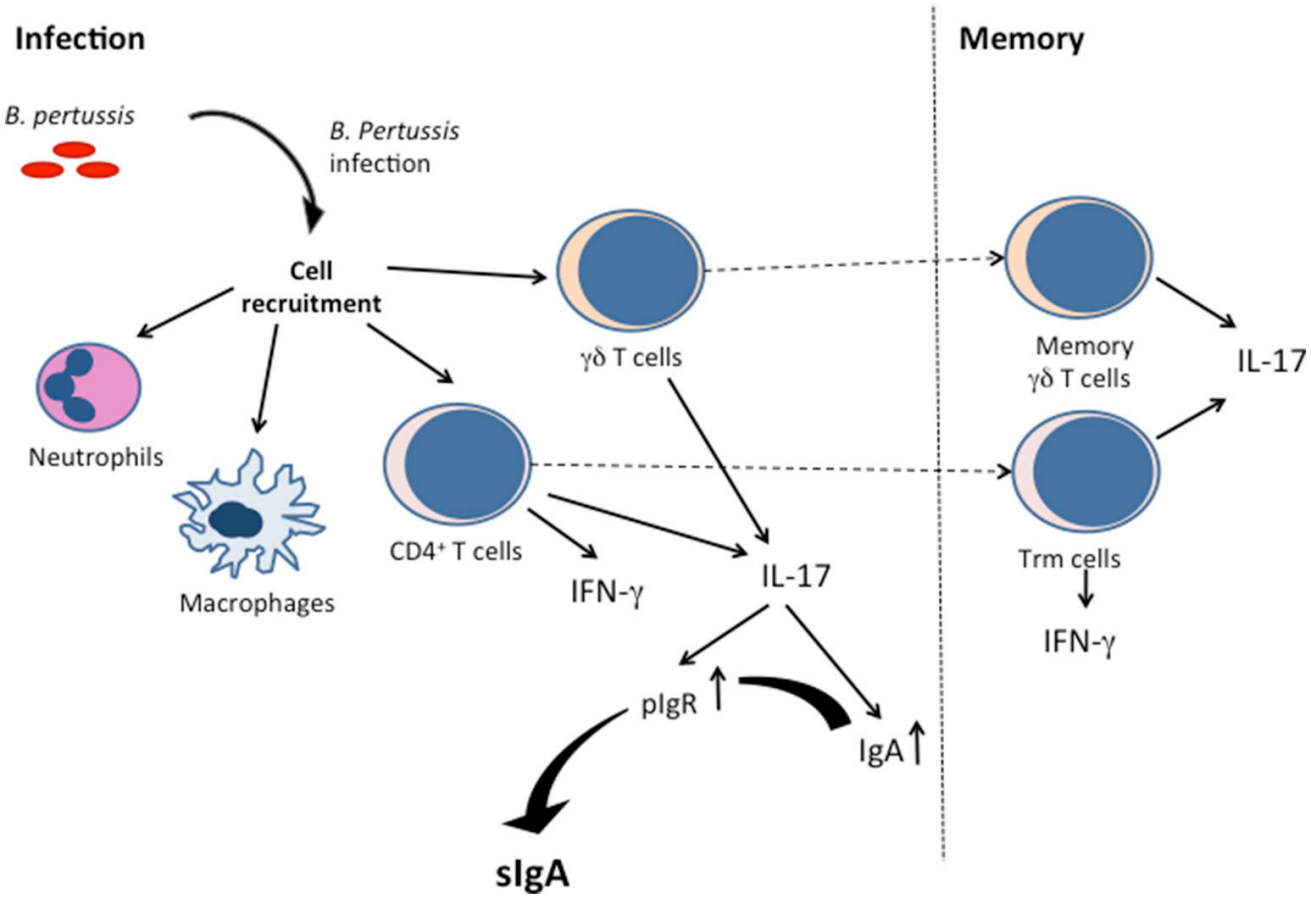
Cellular immune responses and induction of sIgA by *B. pertussis* infection. *B. pertussis* infection induces innate immune cell (such as neutrophils and macrophages) activation, as well as IFN-γ-producing and IL-17-producing T cells. The Th-17 T cells are instrumental for sIgA production. The infection also results in resident memory T cells (γδ and CD4^+^ T cells).

In addition to mucosal antibodies, especially sIgA, *B. pertussis* infection also induces cellular immune mechanisms in the airways (see Figure 1). As such, neutrophils, and macrophages, but also CD4^+^ T cells, and γδ T cells infiltrate the lungs of mice after *B. pertussis* infection (29). Neutrophils may kill phagocytosed bacteria, but their role in early *B. pertussis* clearance from the respiratory tract has not been established (30), although they may be important at later stages of the infection via induced opsonizing antibodies. Furthermore, PTx produced by *B. pertussis* during infection inhibits neutrophil recruitment into the lungs, which may delay the clearance of the organism by opsonizing antibodies (31), suggesting that they are important at certain stages of the infection, and that *B. pertussis* uses its toxin to subvert its neutrophil-mediated elimination. Airway macrophages may also contribute to clearance of *B. pertussis*, as their depletion in mice by the administration of liposome-encapsulated clodronate enhanced the infection despite the influx of neutrophils (32).

For obvious reasons detailed data on local immune cell responses to *B. pertussis* infection in humans are not available, but they have been studied in mice. *B. pertussis*-specific CD4^+^ T cells can be detected in the lungs of mice ~3 weeks post-infection and have been shown to secrete IFN-γ and/or IL-17 (33, 34). IFN-γ-deficient mice show a higher bacterial burden and clear the infection later than wild-type mice (35), indicating an important role of this cytokine in the control of *B. pertussis* infection.

At least some of these *B. pertussis*-specific IFN-γ- and/or IL-17-producing CD4^+^ T cells that can be found in the mouse lungs after infection may be tissue-resident memory T (Trm) cells, as characterized by the expression of CD44, CD69, and/or CD103 and the lack of expression of CD62L. These cells reside only in peripheral tissues and do not re-circulate. When induced by a first encounter with a pathogen at a mucosal site, they respond rapidly to a second infection with the same organism. Wilk et al. (36) have shown that CD4^+^CD69^+^ and/or CD103^+^ T cells are indeed induced in the lungs of mice upon *B. pertussis* infection, and their increase in numbers was associated with *B. pertussis* clearance. These cells persisted for several months in the lung tissues and expanded rapidly and significantly *in situ* upon re-infection. They produced IFN-γ and/or IL-17, and were thus of the Th1 and Th17 type, although most of the cells were of the Th17 type. The specific role of Trm cells can be examined in mice by a treatment with the sphingosine-1-phosphage receptor agonist FTY720 (fingolimod), an inhibitor of lymphocyte migration from the lymph nodes to the mucosal tissues (37). Upon treatment with FTY720, the only T cells present in the mucosa are almost exclusively Trm cells. When mice were treated with FTY720, primary infection with *B. pertussis* was significantly prolonged, whereas the drug had no effect on the course of a secondary infection (36), suggesting that the locally residing T cells developed during the primary infection and rapidly expanding during the secondary infection play a critical role in clearance of the secondary infection. This was confirmed by the adoptive transfer of the lung CD4^+^ T cells from convalescent mice to naïve, sub-lethally irradiated mice, which significantly reduced the bacterial burden after infection of these mice with *B. pertussis*. This was not seen when lung CD4^+^ T cells from naïve mice were transferred.

In addition to CD4^+^ Trm cells, *B. pertussis* infection also leads to the expansion of γδ Trm cells in the lungs of mice (38). Although γδ T cells are usually involved in innate immune responses, they can also mount antigen-specific responses, feature memory-like phenotypes and express CD69 and/or CD103, like CD4^+^ Trm. IL-17-secreting γδ T cells appear in the lungs of *B. pertussis*-infected mice within hours after aerosol exposure, providing an early innate source of IL-17. Their frequency then rapidly declines and rises again 1 week later. The cells of the first wave are predominantly Vγ4^−^γ1^−^ cells, whereas those of the second wave are Vγ4^+^ cells. In the presence of antigen-presenting cells, but not in their absence, these latter cells respond *in vitro* to *B. pertussis* antigens by the secretion of IL-17. Moreover, they further expand upon re-infection with *B. pertussis*, suggesting that the primary infection had induced memory γδ T cells that persist for prolonged periods of time in the lungs. However, to what extend these cells contribute to the control of *B. pertussis* infection has not yet been established.

As these Trm cells are induced by infection but not by acellular pertussis vaccination, their induction may be linked to long-lived immunity, as seen after natural infection in contrast to immunization with pertussis vaccines, especially with acellular vaccines (39). However, recent data indicate that intranasal administration of acellular pertussis vaccines formulated with TLR2 and STING agonists is also very effective in inducing IL-17-secreting Trm cells and long-term protection against nasal colonization by *B. pertussis* (40).

Transcriptomic analyses of *B. pertussis*-infected mouse lungs showed that already 4 h post-infection, the gene expression profile started to change. The changes were maximal 14 days post-infection, after which the profile slowly returned to basal levels (41). As could be expected, acute phase responses and chemotaxis were observed at the early time points, followed by innate immune responses, phagocytosis, complement activation, antigen processing, and presentation, and finally genes involved in the adaptive immune responses at later time points. Infection induced the expression of cytokine and chemokine genes involved in chemotaxis, cell recruitment, and Th1/Th17 responses, as well as genes coding for membrane receptors, including Fc receptors, pIgR, and mucosal homing receptors, B-cell receptor signaling and antibody formation.

Infection by *B. pertussis* also induces local regulatory T cell responses in mice (42). These cells secret high levels of IL-10 and suppress Th-1 responses in the lungs, which may lead to subversion of protective immunity against *B. pertussis* and may also limit inflammatory pathology in the lungs (43).

## Pertussis Vaccination Via Mucosal Routes

Since natural *B. pertussis* infection induces potent mucosal immune responses and sterilizing immunity, in contrast to immunization with currently available vaccines, and since infection provides longer lasting protection than vaccination, it seems logical to use the mucosal, especially the nasal route to vaccinate against *B. pertussis* colonization and disease. Attempts to explore the mucosal routes for vaccination against pertussis have been undertaken in humans and in mice and have initially been focused on the oral route.

### Oral Vaccination Against Pertussis

One to three oral administrations of a killed *B. pertussis* suspension to newborn babies were well-tolerated and able to induce agglutinating serum antibodies (44). It is not known whether this immunization regimen induced mucosal antibodies, especially in the nasal cavity, nor whether it effectively protected these newborns against *B. pertussis* infection and disease. However, in a subsequent study, oral immunization of newborn infants with a whole-cell pertussis vaccine was shown to result in a rise in anti-*B. pertussis* antibody titers in saliva, and these antibody titers were higher in the saliva than in the serum (45), indicating the induction of mucosal immune responses. A study involving more than 20,000 newborns, who received orally 10^12^ killed bacterial cells on days 2, 3, 4, and 5 after birth confirmed that oral immunization with whole-cell pertussis vaccines induces serum and mucosal antibodies in the saliva against *B. pertussis* antigens (46). Although there was a lower frequency of pertussis disease in the orally vaccinated compared to the non-vaccinated children during the first year of life, this difference disappeared after the first year.

With the advent of recombinant DNA technologies, several bacterial vectors that can be administered orally have been engineered to produce individual protective *B. pertussis* antigens, mostly those antigens that are components of the acellular pertussis vaccines, such as PTx, filamentous hemagglutinin (FHA), and pertactin. Live attenuated *Salmonella* strains have been extensively evaluated as recombinant vehicles for the presentation of heterologous antigens to the mucosal immune system by oral administration. The *B. pertussis* pertactin gene has been successfully expressed in a *Salmonella typhimurium aro* vaccine strain (47). Oral vaccination with this strain resulted in a significant decrease of *B. pertussis* burden in the lungs after aerosol infection, as compared to non-vaccinated mice, or mice that had received the non-recombinant *Salmonella* strain. However, antibody levels to pertactin were undetectable after vaccination with the recombinant *Salmonella* strain, but were slightly higher after challenge than for the non-vaccinated mice, suggesting that the vaccination had primed the antibody response. Antibodies against FHA could however be detected after the oral administration of a recombinant *Salmonella dublin aroA* mutant (48). Both anti-FHA serum antibodies, including IgA, as well as anti-FHA IgA in gut wash fluids could be readily detected after feeding mice with the recombinant strain. Another study has shown that feeding of mice with a recombinant FHA-producing *S. typhimurium aroA* mutant or invasive *Escherichia coli* strain resulted in the appearance of anti-FHA IgA in the bronchoalveolar lavage fluids (49).

PTx is a complex multimeric protein composed of five different subunits [for review see (50)]. It is a protective antigen and included in all pertussis vaccines. Monoclonal antibodies directed against the S1 subunit of PTx (PTxA) have been shown to have strong protective potential in mouse models (51). Therefore, the *S. typhimurium aroA* and the invasive *E. coli* strains were also engineered to produce PTxA (52). Oral administration of these strains again resulted in high anti-PTxA antibody titers in the serum and anti-PTxA IgA in the lung lavages. However, the protective potential of these recombinant *Salmonella* and *E. coli* strains against *B. pertussis* challenge had not been addressed in any of these studies. Protection has been evaluated, however, against a recombinant *S. typhimurium aroA* mutant producing all five PTx subunits. Although this strain induced significant anti-PTx antibodies, it failed to protect mice from *B. pertussis* challenge (53).

In addition to attenuated pathogenic bacteria commensal bacteria, such as *Streptococcus gordonii*, have also been tested as delivery vehicles of *B. pertussis* antigens. A recombinant *S. gordinii* strain that presents PTxA on its surface via the transport by the *Streptococcus mutants* major surface protein P1 was found to elicit sIgA against PTx in the saliva and bronchoalveolar lavage fluids of mice upon oral feeding (54). However, again, protection was not assessed in this study.

In addition to mucosal delivery via recombinant bacterial vectors, *B. pertussis* antigens can also be formulated in nanoparticles to stabilize the antigens and to prevent degradation during transit through the digestive tract. When FHA and inactivated PTx were entrapped in poly-lactide-co-glycolide (PLG) nanoparticles and delivered orally multiple times to mice, T-cell responses were induced, as evidenced by IL-5, and low levels of IFN-γ secretion by splenocytes stimulated with FHA or PTx (55). However, the levels of these responses were highly variable between the animals. Orally immunized mice also induced antigen-specific IgA in the lungs, but their levels were not higher than those induced with the same antigens in solution. When the mice were aerosol challenged with virulent *B. pertussis*, a certain level of protection was observed, which was slightly stronger in the mice immunized with the encapsulated antigens. Although these studies provide a proof of concept that oral immunization may induce protective immunity to *B. pertussis*, at least in mice, strong oral adjuvants are certainly needed to enhance these responses.

### Nasal Vaccination Against Pertussis

As *B. pertussis* is a respiratory pathogen, the induction of immune responses in the respiratory tract may be more effective to protect against this pathogen than oral immunization. The protective effect of intranasal vaccination against pulmonary *B. pertussis* infection in mice has already been shown in the 1940s (56, 57), at a time when the first whole-cell vaccines started to come in use and was suggested to be due to both systemic and local immune responses induced by the vaccination. However, intranasal vaccination with whole-cell vaccines provided no protection in the intracerebral challenge model (58), the classical mouse protection assays routinely used for the lot release of whole-cell pertussis vaccines (59). One of the first studies to administer pertussis vaccines via the respiratory tract to humans was published by Gerald Thomas in 1975 (60). He administered a whole-cell preparation by aerosol inhalation into the nostrils of 8 adult volunteers as a single dose. The aerosol vaccination was well-tolerated, better than intramuscular whole-cell vaccination in adults, and increased the IgA levels to *B. pertussis* antigens by ~2-fold in the respiratory secretions, but it did not induce serum antibody responses.

Human nasal vaccination with whole-cell pertussis vaccines were more recently followed up by vaccinating six adult volunteers, who had been immunized in their childhood, with a whole-cell vaccine consisting of 250 μg of *B. pertussis* protein in a 0.5 ml volume, given four times at weekly intervals. The nasal vaccine was overall well-tolerated and induced a significant increase in nasal IgA, as well as systemic IgA and IgG to *B. pertussis* antigens (61). The vaccination did not induce any appreciable level of anti-PTx antibodies, except for one subject, and only a modest increase in nasal anti-FHA IgA in five out of the six individuals. Enhanced T-cell responses could also be measured in these individuals, as evidenced by increased T-cell proliferation to *B. pertussis* whole-cell extracts (62). All six individuals showed a >2-fold increase in T cell proliferation. All vaccines also responded to FHA and four out of the six responded to PTx with a significant rise in T cell proliferation. Although there was no significant correlation between the concentrations of specific serum IgG and T cell proliferation, a significant correlation was found between T cell proliferation, and sIgA in the nasal fluids to whole-cell extracts. Whether intranasal vaccination with whole-cell pertussis vaccines indeed protects humans from *B. pertussis* infection and whooping cough disease remains unknown so far.

Protection against *B. pertussis* infection through nasal vaccination using defined antigens has been evaluated in mouse models. However, purified soluble antigens are usually poorly immunogenic when delivered nasally. Therefore, they have to be combined with mucosal adjuvants to elicit strong antigen-specific sIgA responses. Some of the most potent mucosal adjuvants are cholera toxin (CT) and *E. coli* heat-labile entertoxin (LT). These toxins are A-B toxins, in which the A subunit carries an enzymatic activity whereas the B moiety is responsible for the toxin binding to its receptors. As the CT B subunit has adjuvant properties even in the absence of the A subunit, a hybrid protein was constructed consisting of two copies of PTxA fused to the C-terminal portion of CTA and to CTB (63). This chimeric toxin induced anti-PTx serum IgG and mucosal IgA in the bronchoalveolar lavage fluids, the saliva, and vaginal washes after 6 intranasal immunizations with each 25 μg of the purified hybrid toxin. The serum anti-PTx antibodies were able to neutralize PTx action on Chinese Hamster Ovary cells. When the immunized mice were challenged with virulent *B. pertussis*, the bacterial counts in the lungs were more than 10-fold lower than in the non-vaccinated mice 7 days after challenge.

Instead of genetically fusing *B. pertussis* antigens to CTB or LTB, detoxified CT, or LT can also be mixed with acellular pertussis vaccines to enhance immunogenicity when given nasally. This was shown in a murine study, in which an acellular pertussis vaccine was mixed with genetically inactivated LT and given nasally twice at a 4-week interval (64). This resulted in enhanced antigen-specific serum IgG and sIgA, as well as local and systemic T-cell responses, and was associated with accelerated clearance of *B. pertussis* bacteria from the lungs after challenge.

Interestingly, whereas the addition of enzymatically inactive LT clearly enhanced protective immunity to acellular pertussis vaccine given nasally, this was not the case when whole-cell vaccines were mixed with CT and administered nasally up to four times to mice. Neither the levels of serum IgG or IgA, nor of IgA in saliva or bronchoalveolar lavage fluids to *B. pertussis* extracts were enhanced by the addition of CT (65). Instead, the IgA levels were significantly reduced when CT was given intranasally together with whole-cell pertussis vaccine. This may be due to the fact that whole-cell pertussis vaccines are already potent immunogens and have intrinsic adjuvant activity when given nasally (66) and/or that CT is toxic to the mucosal membrane of the respiratory tract (67). Furthermore, CTB and LTB may cause Bell's Palsy (68), which precludes their widespread use in humans.

However, potent immune responses to *B. pertussis* antigens can also be induced nasally when the vaccines are formulated with different adjuvants. When the cationic polysaccharide chitosan was combined with FHA and genetically detoxified PTx and administered intranasally, high serum IgG, and sIgA levels in lung lavages and nasal washes were observed to both antigens and were considerably higher than when the two antigens were given without chitosan (69). Other nasal adjuvants are onjisaponins from the roots of *Polygala tenuifolia*. When onjisaponins were given nasally together with a pertussis vaccine, a significant increase in serum IgG, and nasal IgA levels to *B. pertussis* antigens were seen compared to the pertussis vaccine alone (70). However, the effect of these adjuvant formulations on protection was not examined in any of the two studies. CpG-containing oligodeoxynucleotides have also been shown to enhance systemic and local antibody responses to PTx, FHA, and pertactin when administered nasally and increased the protection against *B. pertussis* challenge in mice (71), as did the addition of poly[di(sodium carboxylatophenoxy)phosphazene] (72).

More recently, bacterium-like particles (BLP) based on the food-grade bacterium *Lactococcus lactis* have been explored for intranasal vaccination (73). These BLP are generated by the treatment of *L. lactis* with hot acid, which retains the peptidoglycan matrix, and therefore the shape of the organism. When mixed with an acellular pertussis vaccine and administered intranasally, these particles strongly increased the serum IgG titers to the *B. pertussis* antigens and elicited *B. pertussis*-specific IgA responses in the nasal washes. This was associated with a significant level of protection compared to mice that were intranasally vaccinated with the acellular pertussis vaccine in the absence of BLP.

Outer membrane vesicles (OMV) are yet another promising approach for respiratory immunization. Ten years ago, Roberts et al. (74) showed that intransal administration of OMV prepared from *B. pertussis* and containing among others surface antigens, PTx, adenylate cyclase toxin, and lipooligosaccharide, to mice resulted in significant protection against pulmonary colonization by virulent *B. pertussis* upon intranasal challenge. More recently, aerosol vaccination with *B. pertussis* OMV has been shown to provide superior protection against *B. pertussis* challenge than subcutaneous vaccination with the same OMV, especially in the trachea and in the nose (75). Aerosol vaccination with the OMVs led to pulmonary anti-*B. pertussis* IgA, IgA-producing plasma cells, and Th17 cells in the lungs, as well as a rapid induction of pro-inflammatory cytokines and chemoattractants, such as IL-6 and CXCL10. Spray dried OMV with improved stability especially at high temperatures delivered twice by aerosol have also been shown to provide protection against *B. pertussis* colonization of the lungs, trachea, and nasal cavity (76). Importantly, the spray dried formulation was more protective in the nose than the same OMV in a liquid formulation.

Although some of these novel avenues show promise as mucosal vaccine candidates against pertussis disease and infection, none of them have yet entered clinical evaluation. So far, the most effective way to induce protective immunity against *B. pertussis* infection and disease is infection itself, as seen in both mouse and non-human primate studies (16). Furthermore, immunity induced by natural infection is longer lasting than that induced by immunization (39). These observations have led to the concept of live attenuated pertussis vaccines. The first live attenuated vaccine candidate was an *aroA* mutant of *B. pertussis* (77). This strain did not persist in the lung and induced protection against challenge after repeated intranasal administrations. A more recent *aroQ* mutant of *B. pertuss*is persisted longer than the *aroA* mutant and provided protection against challenge already after a single nasal administration (78). A different strategy to engineer a live attenuated pertussis vaccine was based on the genetic elimination or inactivation of PTx, tracheal cytotoxin, and dermonecrotic toxin, which led to the vaccine candidate BPZE1. This vaccine candidate is highly attenuated, even in immune-compromised hosts, yet very immunogenic, and protective in mice after a single intranasal administration [for review see (79)]. Recently it was also shown to be immunogenic in baboons and to elicit high levels of IgG and IgA against PTx, FHA, and pertactin and to protect these baboons from severe pertussis induced by challenge with a very high dose of a highly virulent *B. pertussis* clinical isolate (80). Furthermore, upon challenge with this highly virulent isolate it reduced the overall bacterial burden by 99.998% over the non-vaccinated animals. BPZE1 is the most advanced novel pertussis vaccine candidate and has successfully completed phase I trials, where it was found to be safe in young human adults, able to transiently colonize the human nasopharynx, and to induce antibodies to PTx, FHA, pertactin and fimbriae after a single nasal administration (81). This vaccine candidate is currently entering a clinical phase II trial. Human monocyte-derived dendritic cells *in vitro* stimulated with BPZE1 were shown to polarize T cells toward a Th17 response (82).

In mice a single nasal administration of BPZE1 protected against challenge colonization of both the lungs and the noses, whereas two administration of 1/5 of a human dose of an acellular vaccine only induced protection in the lungs, but not in the nose (27). In addition, only BPZE1 induced *B. pertussis*-specific sIgA in the nasal cavity, and transfer of the nasal IgA was able to protect recipient mice against nasal colonization after *B. pertussis* challenge. These protective nasal IgA were not produced when poly-Ig-receptor-deficient mice were vaccinated with BPZE1, indicating that they were genuine sIgA. Furthermore, IgA-deficient or poly-Ig-receptor-deficient mice were much less protected by BPZE1 against nasal colonization by virulent *B. pertussis*, illustrating the critical role for IgA, and its secretion into the nasal cavity in protection. Moreover, protection against *B. pertussis* infection of both the lungs (83) and the noses (27) was long lived. BPZE1 also induced CD4^+^CD69^+^CD103^+^ Trm cells in the nasal mucosa of mice, and these cells produced high levels of IL-17, but also appreciable levels of IFN-γ. The important role of IL-17 in the protection against nasal colonization by virulent *B. pertussis* was demonstrated by the fact that IL-17-deficient mice were no longer protected by intranasal vaccination with BPZE1 and also produced lower levels of sIgA than the wild-type mice after BPZE1 administration. Thus, BPZE1 protects mice against nasal infection by virulent *B. pertussis* via an IL-17-dependent sIgA-mediated mechanism.

## Concluding Remarks

*B. pertussis* is a strictly respiratory pathogen, mainly found attached to the ciliated cells of the upper respiratory tract. Yet, routine vaccination is done parenterally, inducing circulating antibodies, and systemic cell-mediated immunity. Local mucosal immunity is not induced by the current vaccination regimens, which is likely the main reason why pertussis vaccination fails to control *B. pertussis* infection and only induces at best modest herd immunity. The fact that in many countries, in which high coverage with acelullar vaccines containing pertactin is achieved, pertactin-deficient *B. pertussis* strains are increasingly isolated (84–86), most likely due to vaccine pressure, suggests that some degree of anti-infection immunity is induced by some acellular vaccines. However, this is not sufficient to effectively control *B. pertussis* circulation even in highly vaccinated populations. It is most likely that local immunity is required for effective protection against infection by *B. pertussis*. Potent local antibody and T-cell responses are indeed induced upon natural infection in humans and experimental infection in mice and non-human primates. Since infection induces sterilizing immunity, these responses are likely to play a critical role in infection control. Several attempts have been made in both humans and animal models to induce local immunity by vaccination via the oral or nasal route. However, none of them have reached the stage of mass vaccination regimens. With a deeper understanding on protective local immunity, as it has emerged over the years, mucosal, especially nasal vaccination has recently attracted interest again, especially by using novel approaches, such as nasal delivery of live attenuated *B. pertussis* vaccines. One such candidate, BPZE1, is currently in clinical development and shows promise for providing durable local immunity and improved control of pertussis. It will of course be important to know whether nasal vaccines such as BPZE1 will protect against infection by the various *B. pertussis* clades currently circulating, as allelic variations of protective antigens have been proposed to contribute to the current pertussis resurgence in several countries. Furthermore, the effect of maternal antibodies transmitted to the offspring on immunity induced by mucosal vaccines is not yet known. This is of particular importance, as maternal immunization against pertussis is now recommended in several countries, based on the high effectiveness of this strategy to protect infants against severe pertussis in the first months of life (87).

## Author Contributions

LS prepared the first draft of the paper and critically revised the final draft. CL prepared the final draft of the paper.

### Conflict of Interest Statement

The employer of CL holds patents concerning the live attenuated pertussis vaccine BPZE1, and the patent portfolio has been licensed to ILiAD Biotechnologies. The remaining author declares that the research was conducted in the absence of any commercial or financial relationships that could be construed as a potential conflict of interest.
